# A multimodal and signals fusion approach for assessing the impact of stressful events on Air Traffic Controllers

**DOI:** 10.1038/s41598-020-65610-z

**Published:** 2020-05-25

**Authors:** Gianluca Borghini, Gianluca Di Flumeri, Pietro Aricò, Nicolina Sciaraffa, Stefano Bonelli, Martina Ragosta, Paola Tomasello, Fabrice Drogoul, Uğur Turhan, Birsen Acikel, Ali Ozan, Jean Paul Imbert, Géraud Granger, Railane Benhacene, Fabio Babiloni

**Affiliations:** 1 Dept. of Molecular Medicine, Sapienza University of Rome, Piazzale Aldo Moro, 5, 00185 Rome, Italy; 2 BrainSigns srl, Lungotevere Michelangelo 9, 00192 Rome, Italy; 3 IRCCS Fondazione Santa Lucia, Neuroelectrical Imaging and BCI Lab, Via Ardeatina, 306, 00179 Rome, Italy; 4 Dept. of Anatomical, Histological, Forensic & Orthopedic Sciences, Sapienza University of Rome, Piazzale Aldo Moro, 5, 00185 Rome, Italy; 5 DeepBlue srl, Piazza Buenos Aires 20, 00185 Rome, Italy; 6 École Nationale de l’Aviation Civile, 7 Avenue Edouard Belin, 31000 Toulouse, France; 7 Eskişehir Technical University, The Faculty of Aeronautics and Astronautics. Iki Eylul Campus, 26555 Tepebaşı/ Eskişehir, Turkey; 8 Kastamonu University, School of Civil Aviation, Kuzeykent Campus, 37200 Kastamonu, Turkey; 9 College of Computer Science and Technology, Hangzhou Dianzi University, Hangzhou, China; 10 EUROCONTROL, Rue de la Fusée 96, 1130 Brussels, Belgium

**Keywords:** Stress and resilience, Biomedical engineering

## Abstract

Stress is a word used to describe human reactions to emotionally, cognitively and physically challenging experiences. A hallmark of the stress response is the activation of the autonomic nervous system, resulting in the “fight-freeze-flight” response to a threat from a dangerous situation. Consequently, the capability to objectively assess and track a controller’s stress level while dealing with air traffic control (ATC) activities would make it possible to better tailor the work shift and maintain high safety levels, as well as to preserve the operator’s health. In this regard, sixteen controllers were asked to perform a realistic air traffic management (ATM) simulation during which subjective data (i.e. stress perception) and neurophysiological data (i.e. brain activity, heart rate, and galvanic skin response) were collected with the aim of accurately characterising the controller’s stress level experienced in the various experimental conditions. In addition, external supervisors regularly evaluated the controllers in terms of manifested stress, safety, and efficiency throughout the ATM scenario. The results demonstrated 1) how the stressful events caused both supervisors and controllers to underestimate the experienced stress level, 2) the advantage of taking into account both cognitive and hormonal processes in order to define a reliable stress index, and 3) the importance of the points in time at which stress is measured owing to the potential transient effect once the stressful events have ceased.

## Introduction

### Stress in air traffic management

The air traffic management (ATM) system is currently going through a major restructuring process, covering the entire architecture of process, personnel, and management environments. The increase in the level of automation as support tools for air traffic control (ATC) activities is one of the main technological challenges which will be faced in the future^1,2^. Automation, digitalisation of information and virtualisation of the ATC operations will require a transformation of tasks and working methods. For example, with the prospect of an increasing in the volume of air traffic to be managed, controllers will be expected to delegate more activities and decisions to automation. In ATC, high demand is usually associated with a high mental workload and level of responsibility, while stress is linked to available time, team support, adherence to procedures and control over the planning and execution of tasks. The concepts of mental workload and stress are often used ambiguously, especially because of their relationship with performance when they reach very low or high values. In such cases, performance drops dramatically. However, certain levels of mental workload and stress allow an operator to maintain a proper level of engagement and consequently achieve a high level of performance^3^. Moreover, in some theories, stress is considered a component of mental workload^4^. It is, however, important to note that reactions to stress can also occur when mental workload is low, as is the case in monotony and isolation, and alternatively high mental workload can appear during low stress conditions, as when processing unfamiliar information or solving complex problems where there are no time constraints. The main confusion between the two conceand temporal demand imposed by a task (e.g. the amount of information to be processed or number of tasks to be performed simultaneously)^3^. The concept of *stress* can be seen as the result of one or more stressors affecting the surrounding environment (e.g. noise, a crowed working place, room temperature), work-related factors (e.g. technology failure, time pressure, miscommunication), or the subjective evaluation of the situation (e.g. availability of appropriate resources to handle the situation in question)^4,5^. In other words, the main differences between mental workload and stress can be summarised on the basis of the following aspects^4,6–9^:

- The neurophysiological response to mental workload usually results in more focused attention and improved efficiency. By contrast, the response under stress may even be dysfunctional, causing distraction and reduced efficiency.
- The neurophysiological response to mental workload is limited to the period during which the task has to be executed, and it returns to the baseline in due course once the task has been completed. Under stressful conditions, however, the responses will persist even after the event causing the stress is over.
- Under mental workload, a situation is experienced as a challenge and the user is positively motivated, resulting in feelings of accomplishment and ‘positive fatigue’. On the contrary, under stressful conditions, the user can feel threatened, resulting in strain and negative emotions.

In line with this, the major cause of stress in ATC is not only the combination of high mental workload with time pressure but also all situations which reduce control over one’s activity or require one to change plans. Air traffic controllers (ATCOs) are well trained to cope with stress, but prolonged exposure to intense pressure is likely to give rise to serious consequences and increase the risk of error. As a result, stress management abilities are important skills to be monitored and maintained. In this connection, Commission Implementing Regulation (E\U) 2017/373 (GM1 ATS.OR.310) acknowledges stress as an important safety problem and establishes the requirements to be met by EU air navigation service providers (ANSPs) to prevent and mitigate the negative effects of stress on ATCOs in order to ensure the safety of air traffic^10^. For example, the Regulation requires ANSPs to develop and maintain a policy for the management of ATCO stress. The Regulation recommends that stress intervention/mitigation/prevention practices be adopted, including stress management training for all levels of employees, staff support mechanisms, and completion of regular risk assessments^10^. Similarly, the Commission Regulation (EU) No 2015/340 on the licensing and medical certification of ATCOs explicitly includes stress-related symptoms in the medical conditions to be evaluated in order to assess the fitness of ATCOs (ATCO.MED.B.060 Psychology). At the same time, the job of a controller includes a high level of responsibility in terms of both its social and its economic impact^11^. In this connection, the current study aimed to develop a neurophysiological index in order to characterise a controller’s stress response during the execution of ATC activities, to be potentially used during training or testing activities in order to better manage and arrange volumes and sectorisation of airspace^12^, and finally provide an objective measurement to support ANSPs in dealing with controller stress management and EU regulations.

### Neurophysiology of stress

Stress response in humans is mediated by a complex and interconnected neuroendocrine cellular and molecular infrastructure which constitute the stress system and is located in both the *central nervous system* (CNS) and the autonomous nervous system (ANS)^13^. A hallmark of the stress response is the activation of the *hypothalamo-pituitary-adrenal* (HPA) axis and subsequent secretion and release of hormones. Two main classes of stress hormones can be identified, namely *glucocorticoids* (i.e. cortisol), and *catecholamines* (i.e. adrenaline and noradrenaline). When these two hormones are released in response to stressful events, they trigger the *fight-freeze-flight* response^14,15^. In such cases, users may experience increased *heart rate variability* (HRV), sweating, and blood pressure^16–18^. Thus although the CNS is directly or indirectly involved in preserving homeostasis, the whole body acts in orchestrating the stress response. To investigate these aspects, Koelsch *et al*.^19^ measured the effects of stress on several mediators. They noted that Sodium (Na) and ACTH showed a fast and phasic response, whereas cortisol reached a maximal value more than 15 minutes after the stress test was terminated, thus showing a much slower response and a gradual decrease over time. These results can be explained by the fact that both ACTH and Na are released following electrical (i.e. neuronal) signals, whereas cortisol is secreted humorally^19^. In other words, the releasing of adrenergic stimuli in the blood stream persists even after the stressor is terminated owing to the presence of catecholamines. As a main consequence of this, hormonal processes (measured by the *galvanic skin response* - (GSR) and *electrocardiogram* (ECG) autonomic signal) may take longer to recover normal activations than cognitive processes (measured by *electroencephalography* – EEG). These residual effects will result in a transient effect over time, and they could also make the user underestimate the stress experienced and lead to hazardous situations, especially in high-risk environments. For example, in operational contexts such as ATM, it is commonplace to have to deal with very stressful factors such as unexpected events, technical failures and time pressure. In other words, when asked to rate the perceived stress level, users are likely to underestimate the actual stress state and they may be inclined to commit mistakes and errors.

### State of the art on stress assessment in ATM

Air traffic control has been classified by the U.S. Department of Labor as the fourth most stressful job^20,21^. The majority of studies related to ATM contexts are focused on task complexity and mental workload rather than investigating the impacts and consequences of stress on controllers^22–34^. Stress responses are characterised by the onset of mental and physical alterations. For example, in the presence of stressors, brain activity in the beta EEG band increases^35^, and there is asymmetry of brain activations, especially across the frontal brain areas^36^. Moreover, it has been widely demonstrated that components of the GSR and ECG signals modulate with stress^3,37–39^ and could thus be good candidates for stress assessment together with EEG parameters. In particular, the *skin conductance level* (SCL) and *skin conductance response* (SCR) components of the sweat glands, and the *heart rate* (HR) and *heart rate variability* (HRV) derived from the heart activity were considered^40^. However, the point to be addressed in the present work is *how to select the most appropriate and reliable stress measurements*. Most studies generally employ subjective measurements (e.g. self-reports) in order to evaluate a user’s stress level, such as Stressful Life Experiences Screening (SLES)^41^, the Stress and Adversity Inventory (STRAIN)^42^, the Life Events Checklist for DSM-5 (PCL-5)^43^, the Stanford Acute Stress Reaction Questionnaire (SASRQ)^44^, the Inventory of Psychosocial Functioning^45^, the World Assumptions Scale (WAS)^46^, or the Stress Arousal Scale (SAS4)^47^.Subjective measurements, however, have many limitations. For example, while filling in the questionnaire, the execution of the tasks must be interrupted, and they are highly operator-dependent. As a direct consequence of this, subjective measurements cannot be used for real-time assessments, because they interfere with the execution of the tasks and are likely to be biased by the user’s mood, feeling, and surrounding environment or context^3,48^.Multivariable approaches, such as neurophysiological measurements, have therefore been proposed in order to obtain a broad objective measurement of the stress response, thus considering multiple reactions triggered by the stressors. However, we identified three main limitations among the most recent studies. Firstly, certain studies investigated only mental stress (i.e. analysing only the EEG signal) without taking into account hormonal responses related to autonomic signals^49–51^, whilst others focused only on acute emotional stress (e.g. ECG and GSR), thus not considering the impact of stress on cognitive processes^22,49–55^. In our study, we sought the advantages of considering both the cognitive and the hormonal aspects at the same time. Secondly, even though some studies recruited professional controllers, they were conducted in controlled contexts and with laboratory tasks (e.g. the Stroop task, or Montreal Imaging Stress)^56–60^. In our study, the controllers dealt with realistic ATM tasks in ecological settings. Thirdly, studies employing multimodal and machine-learning approaches did not consider the importance and meaning of the selected neurophysiological features^58,61–63^. In fact, they usually made blind selections from a very large set of features in order to achieve high-classification accuracy. Such accuracy, however, cannot be directly associated with changes in the investigated mental state (i.e. stress), but possibly with confounds such as the influence of other mental states or with specific events throughout the tasks. Our approach aimed to avoid this effect. In particular, we initially identified the neurophysiological parameters linked to stress, and then employed a machine-learning algorithm on the final feature set in order to select the most significant features to finally define the stress model. Consequently, the proposed study aimed to (i) better characterise stress in ATM contexts by adopting a multimodal approach and neurophysiological measurements, (ii) investigate the impact of stress on controller performance and self-awareness, and (iii) finally propose a *stress index* in order to objectively assess the stress experienced by controllers in the ATM scenario considered. For this purpose, sixteen ATCOs were asked to deal with a realistic ATC radar simulation while their brain and autonomic signals were collected (by EEG and by ECG and GSR respectively). The experimental hypotheses were that i) stressful events would cause a significant neurophysiological response, ii) stress would impair an ATCO’s stress perception and efficiency, and iii) the impact of stressful events would have a transient effect even after they are over.

## Material and Methods

### Participants

Informed consent for both participation in the study and publication of identifying information/images in an online open-access publication was obtained from sixteen ATCOs after the study, which was approved by the local institutional ethical committee of the Eskişehir Technical University (Eskişehir, Turkey), had been explained. The group was selected with the aim of having a homogeneous sample in terms of gender (all males), age (23.8 ± 1.3), and background skill level (all the participants were at the end of the training period and thus had the same rank and level of ATM operational training). The experiment was conducted following the principles outlined in the Declaration of Helsinki of 1975, as revised in 2000.

### Air traffic management simulation

**Figure 1:**
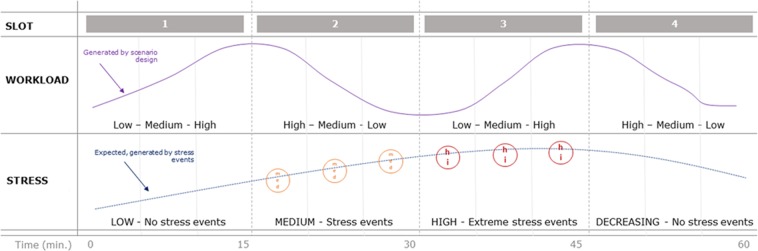
The ATM scenario was designed from real traffic samples normally used in the Eskisehir Technical University training programme, and was modified in accordance with the objectives and needs of the study. The simulation mainly consisted in managing realistic air traffic for a total duration of 60 minutes. Four slots (SLOT#1-SLOT#4) of 15 minutes were identified depending on the stress level (low stress, medium stress, high stress). Within each slot, three five-minute conditions were identified depending on the three workload levels (LOW, MEDIUM, HIGH) for a total of twelve five-minute phases designated LWL1, MWL1, HWL1, LWL2, MWL2, HWL2, LWL3, MWL3, HWL3, LWL4, MWL4, HWL4. The triplets of medium (orange circles) and high (red circles) stressful events were inserted in phases with the same complexity level in order to counterbalance potential biases in the stress assessment due to the different workload demands.

The ATM scenario was designed by a pool of experts from the Eskişehir Technical University (Eskişehir, Turkey), the École Nationale de l’Aviation Civile (ENAC, Toulouse, France), and EUROCONTROL (Brussels, Belgium). In particular, the ATM scenario was designed from real traffic samples normally used in their training programmes, and it was modified in accordance with the objectives and needs of the study. The adjustments, in terms of workload demand, mostly concerned the routes and air traffic within the airspace sectors (adding or changing them), whereas the borders of the sectors were not changed. The air traffic adjustments consisted in rescheduling flights and changing *flight levels* (FLs), in terms of *numbers of aircraft*, *traffic geometry* and *numbers of conflicts*, with the aim of defining different air traffic complexity levels, namely *low* (L), *medium* (M) and *high* (H) on average (but with changing shapes over time), and simulating realistic transitions between such ATC conditions. Moreover, six stressful events were designed, taking into account feedback derived from specific workshops at which professional ATCOs, instructors and human factor experts had identified the most common and worst events/conditions/situations inducing high stress and/or high workload (see the “*stressful events*” section for more details). In addition, since mental workload and stress are closely interrelated^3^, we inserted the triplets of *medium-* and *high-*stress events within phases having the same complexity in order to counterbalance potential biases due to the different workload demands in the overall stress assessment (Fig. 1). In other words, the ATM scenario was designed to mainly differ mainly by the presence and intensity of stressful events, and counterbalance other factors such as workload. The overall experimental protocol consisted in dealing with a highly realistic ATC radar control scenario of 60 minutes within simulated Istanbul airspace. The simulation was managed by volunteer ATC graduates (hereinafter designated *ATCOs*) who had successfully completed their training. In order to stress the ATCOs, specific stressful events were designed and inserted in the ATM scenario. In particular, the simulation consisted mainly of four 15-minute slots (SLOT#1-SLOT#4). Each slot was designed with three workload levels (low – LWL_*i*_, medium – MWL_*i*_ and high – HWL_*i*_, where *i* = 1, 2, 3 or 4 depending on the related slot) in order to counterbalance effects on the stress evaluation. In particular, within SLOT#1 and SLOT#4 there were no stressful events, and they were therefore designated *low-stress* phases. In SLOT#2 (*medium stress*), three 20-second medium-stress events (SM1, SM2, and SM3 – orange circles in Fig. 1) were inserted, while in SLOT#3 (*high stress*) there were three 20-second high-stress events (SH1, SH2, and SH3 – red circles in Fig. 1). Detailed descriptions of these events are given in the *stressful events* paragraph.

Some of the stressful events required control of the traffic by giving instructions to change flight levels and headings and maintain safety separation minima between air traffic, especially in certain conflicted points within the airspace being considered. These separation decisions and instructions had to be taken/given by the controllers, who were responsible of all traffic in their airspace sector. In order to ensure international safety standards (i.e. horizontal separation of 5 nautical miles (NM) and vertical separation of 1000 feet (ft), the ATCOs were asked to separate air traffic both vertically and/or horizontally. *Vertical* separation means that controllers could manage the flight levels of conflicted traffic by clearing appropriate new flight levels for suitable air traffic. *Horizontal* separation means that controllers had to ensure safe separation minima by issuing clearances and/or instructing appropriate aircraft which were in conflicting over a point or in an airway in the airspace to change speed and/or direction.

### Data acquired in the experiment

**Figure 2:**
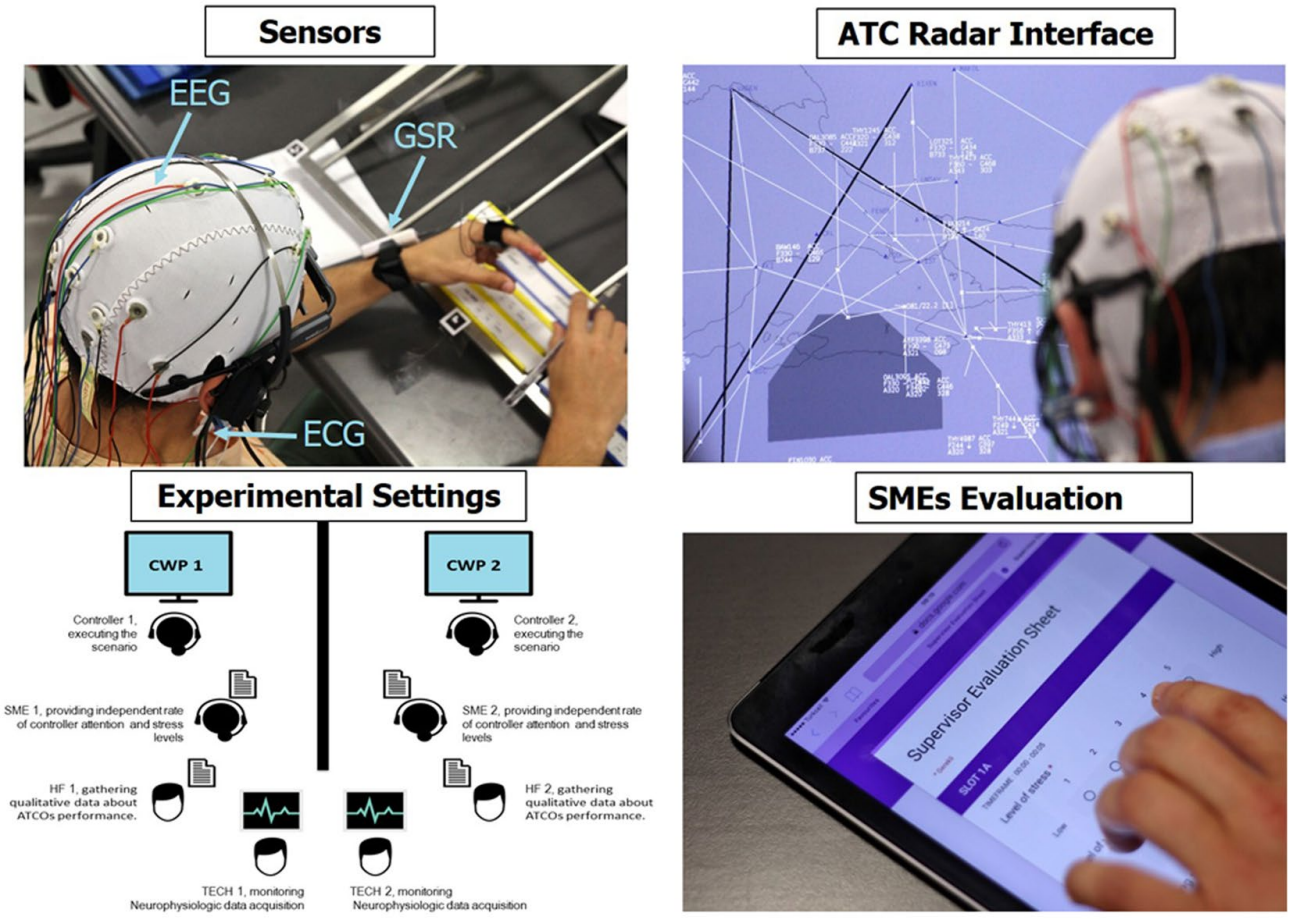
The controllers were asked to deal with the realistic ATM scenario under ecological settings. During the whole experiment, the ATCOs’ neurophysiological signals (EEG, ECG, and GSR) and stress perception were gathered. In addition, SMEs provided ratings about how well the controllers were managing the air traffic (Efficiency) and about the stress under which the ATCOs were working.

While dealing with the ATM scenario, the controllers’ EEG, GSR, and ECG signals were gathered continuously. In addition, the controllers were asked to provide their stress perception by reading out a value between 1 (*no stress*) and 5 (*very high stress*) over the radio when requested by the supervisors (every 5 minutes) in order to identify any differences between perceived and experienced stress (Fig. 2). Before the execution of the experiment, the ATCOs were asked to keep their eyes closed and remain relaxed for a minute (OC condition). Two pseudo-pilots were also involved in the experiments, and they sat in a separate room, playing the role of real pilots flying the aircraft under ATCO control and communicating with them via the proper radio sector frequencies. Furthermore, during the execution of the ATM scenario, *subject-matter experts* (SMEs) were sitting behind the ATCOs to rate how the controllers were managing the air traffic (*Efficiency*), and the stress levels under which the ATCOs were working by filling in the 5-scale questionnaire (Fig. 2).

### Data analysis assumptions

Most of the classification algorithms assume that the individual signal samples are independent of one other. Conceptually, the most commonly used and heavily studied assumption is the *independent and identically distributed* (iid) sample dataset^64–66^. The *independence* assumption states that the sample values are all mutually independent, that is, no sample values depend on any other sample values. However, the sample independence assumption does not mean that the individual components of a vector are independent. The *identically distributed* assumption simply states that all the samples are drawn from the same probability distribution^66^. Most classifier-learning algorithms assume that the training data is *iid*. Nevertheless, this assumption is commonly violated in many real-life problems where sub-groups of samples exhibit a high degree of correlation amongst both features and labels, or where control over the data gathering process is not complete. Consequently, in recent years, learning with dependent samples has been investigated^67–71^, and most recent studies employ *data augmentation* techniques to enrich the training dataset, such as *oversampling*, *data warping*, *overlapping*, and *generative adversarial networks* (GANs)^72–75^. In accordance with these recent works, we used the *overlapping* and *oversampling* methods in our study in order to ensure a high number of observations for the classification algorithm, and to increase the samples of datasets when the classes were unbalanced. Chawla *et al*.^76^ in fact demonstrated how oversampling the minority class allows better classification performance to be to obtained than by only undersampling the majority class (see the “*Data Fusion*” paragraph for more details).

### Stressful events

The stressful events were designed to modulate the status of the controllers while they were managing the air traffic. In particular, six 20-second stressful events were inserted in the ATM scenario: three events to induce *medium* stress were inserted in SLOT#2, and three events to induce *high* stress were inserted in SLOT#3. These events were designed by analysing feedback from professional controllers and instructors gathered together at a workshop with the aim of identifying the most stressful events in air traffic control rooms. Detailed descriptions of these events are reported below.

#### High complexity area conflict (*medium stress*)

Aircraft appeared on the radar screen were approaching one other through the centre of the airspace. ATCOs had to manage vertical and horizontal separation between aircraft. The response and conflict resolution time were very limited relative to aircraft speed, including departing, arriving and especially opposite traffic, causing high complexity. Safety objectives and time pressure induced feelings of stress.

#### Conflict detection due to transponder Mode C failure (*medium stress*)

Transponder-based altitude (i.e. flight level) information was lost from the CWP (controller working position) perspective for short periods of time (20 seconds). Suddenly, an aircraft suffered Mode C failure, and it appeared on the radar screen at the same flight level of other traffic, causing a traffic conflict detection system alert as indicated by a change in the label colour to red and flashing to attract the controller’s attention. This was an unusual and unexpected situation for the controller who was managing and monitoring the organised traffic.

#### Social pressure (*medium stress*)

To simulate social pressure and distraction, a technician stood beside a controller and started playing with a mobile phone for 20 seconds, making it ring loudly. This external distracting affect created social pressure on the controllers, causing distraction and stress^77,78^.

#### Radio noise (*high stress*)

One of the aircraft’s radio communication systems suddenly created noise, resulting in very poor communication with the pilot and difficulty in understanding and providing commands. This situation resulted in extra effort for the controller, who has to monitor and manage all the traffic. After 20 seconds, the technical problem disappeared and the controller could continue the normal communications.

#### Emergency descent (*high stress*)

In the centre of the airspace, an aircraft suddenly declared an emergency descent. This unexpected operation may generally be caused by either technical or security problems in the air traffic, since it can cause crossing and conflicting with other air traffic. A controller has to inform all pilots in proximity positions and instruct them to take evasive action. This can induce great time pressure and stress, since air traffic safety has to be ensured in a complex environment.

#### Radar images lost (*high stress*)

The radar images on screen suddenly disappeared. This meant that the controllers could not monitor any traffic in their area of responsibility but could switch to paper strips only. This could cause a loss of situational awareness and considerable stress for a controller, who needs all the information about aircraft, supporting tools, and safety and efficiency measurements in order to be able to handle the air traffic. After 20 seconds, the radar images came back and the controller was able to recover control of the air sector and traffic.

### Brain activity recording and pre-processing

The EEG was recorded by a digital monitoring system (BEmicro system, EBNeuro S.p.A., Italy) with a sampling frequency of 256 Hz. All 16 EEG electrodes (Fpz, AFz, AF3, AF4, Fz, F3, F4, Cz, Pz, P3, P4, POz, PO3, PO4, O1, and O2) were referred to both earlobes, grounded to the *FCz* channel, and their impedances were kept below 10 kΩ. The EEG signal was initially band-pass filtered with a 5^th^-order Butterworth filter (high-pass filter: cut-off frequency fc = 1 Hz; low-pass filter: cut-off frequency fc = 40 Hz). The *Fpz* channel was then used to remove eye-blink contributions by the REBLINCA algorithm^79,80^. This method allows the EEG signal to be corrected without losing data. For other sources of artifacts (e.g. environmental noise, user movements, etc.) specific procedures of the EEGLAB toolbox^81^ were employed. The EEG dataset was firstly segmented into epochs of 2 s through moving windows shifted by 0.125 s^82^. This windowing was chosen with the compromise of having both a high number of observations, in comparison with the number of variables, and in order to respect the condition of stationarity of the EEG signal^83^. This is in fact a necessary assumption in order to proceed with the spectral analysis of the signal. The EEG epochs with the signal amplitude exceeding ±100 μV were marked as artifacts (*threshold criterion*). Each EEG epoch was then interpolated to check the slope of the trend within the epoch considered (*trend estimation*). If such a slope was higher than 10 μV/s, the epoch considered was marked as an artifact. Finally, the signal sample-to-sample difference (*sample-to-sample criterion*) was analysed: if the difference, in terms of absolute amplitude, was higher than 25 μV, i.e. if there was an abrupt (non-physiological) variation, the EEG epoch was marked as an artifact. At the end, the EEG epochs marked as artifacts were removed from the EEG dataset, with the aim of obtaining an *artifact-free* EEG dataset. The *power spectral density* (PSD) was calculated for each EEG channel and for each epoch using a Hanning window of the same length of the epoch considered (2-second length, i.e. 0.5 Hz of frequency resolution). The EEG frequency bands were then defined for each participant according to their *individual alpha frequency* (IAF) value^84^. Since the experimental settings were highly realistic, the risk of collecting general noise-related data can be high. In order to deal with this critical problem, we proceeded as follows:

- Firstly, we employed gel-based electrodes in order to ensure low impedance (i.e. 10 kΩ) and stable values over the entire experimental protocol (i.e. about 60 minutes) and to limit recording of noise due to external interferences.
- Secondly, every 15 minutes we checked the electrode contacts, especially the reference and ground electrodes, and the impedance values.
- Finally, we employed advanced signal processing techniques, starting with a conservative method (i.e. correcting the data through the REBLINCA) and then in a robust way (i.e. removing the epochs which could not be corrected and reported unusual trends within the time window considered). In this regard, the average number of epochs removed from the dataset was 19.7 ± 8.5% (mean ± standard deviation).

### GSR recording and pre-processing

The GSR was recorded with a sampling frequency of 100 Hz using a Shimmer3 GSR + unit (Shimmer sensing, Ireland) by means of two electrodes on the index and middle fingers of the non-dominant hand. The GSR was firstly down-sampled to 25 Hz and then processed by using the *Ledalab* suite^85^, a specific open source toolbox implemented within MATLAB for GSR processing. *The continuous decomposition analysis*^86^ was applied in order to estimate the *tonic* (SCL) and the *phasic* (SCR) components^87,88^. The SCL is the slow-changing part of the GSR signal, mostly related to the global arousal of the participant, whilst the SCR is the fast-changing part of the GSR signal which occurs in relation to single stimuli reactions^37^. The GSR components were calculated with a different time resolution, with respect to the EEGs, owing to the amount of data necessary for their estimation. In this regard, as stated above, a time resolution of 2 s is appropriate to ensure the stationarity of the EEG signal^89,90^. Estimation of the GSR and ECG parameters, however, requires a time window long enough to detect the variations considered and/or achieve a specific frequency resolution for the estimation of the spectral components, such as the low-frequency (LF) oscillations of the HRV. For example, the SCL and SCR components require a time-window of 5 s^91^, whilst the spectral analysis of the ECG signal, i.e. the HRV estimate, needs a time window of at least 30 s^92^. Consequently, owing to these differences in the lengths of the time window all the neurophysiological parameters considered were averaged every 30 seconds (see the *Data Fusion* sections for more details) before the feature merging step with a moving windows of 0.5 s in order to obtain a high number of observations for classification purposes.

### ECG recording and pre-processing

The ECG signal was recorded with a sampling frequency of 256 Hz by means of an electrode fixed on the chest of the participant, and referred to the potential recorded at both the earlobes. First, the ECG signal was filtered using a 5th-order Butterworth band-pass filter (high-pass filter: cut-off frequency fc = 5 Hz; low-pass filter: cut-off frequency fc = 20 Hz) in order to reject the continuous component and the high-frequency interferences, such as that related to the mains power source. At the same time, the purpose of this filtering was to emphasize the QRS process of the ECG signal, since it has been demonstrated that most of the QRS energy is approximately included between 5 and 15 Hz^93,94^. The following step consisted in measuring the distance between consecutive R peaks (i.e. each R peak corresponds to a heartbeat) of the ECG signal in order to estimate *heart rate* (HR) values. In this regard, the *Pan-Tompkins* algorithm^95^ was employed for the HR estimate. Other artifacts of the HR signal were automatically corrected using the HRVAS suite^96^, an open-source toolbox implemented in MATLAB. Finally, a spectral analysis of the HR signal was performed to estimate the HRV using the Lomb-Scargle periodogram. This method has been demonstrated to produce much more accurate estimates of the PSD than *fast fourier transform* (FFT) methods for typical HR data^97^. Since the HR data is unevenly sampled, another advantage of the Lomb-Scargle method is that it can be used without the need to resample and de-trend the RR data^98^ in contrast with FFT-based methods. Thirty-second windows were considered in order to obtain a frequency resolution of 0.033 Hz and allow the analysis of the characteristic HRV frequency sub-bands. In particular, in line with the scientific literature^99^, the PSD of the HR signal was computed over the low (LF: 0.04–0.15 Hz) and the high frequencies (HF: 0.15–0.4 Hz), and the HRV parameter was calculated as the ratio *LF/HF*.

### Overall description of the analyses

In the first phase of the work, the various neurophysiological parameters (i.e. PSD, HR, LF/HF, SCL, and SCR) were analysed individually by statistically comparing the ATM phase with no stressful events, i.e. SLOT#1, and the ATM phase with the high-stress events, i.e. SLOT#3. Both ATM phases were similar not only in terms of absolute workload levels but also in terms of the sequence of workload levels: easy → medium → hard. Consequently, the purpose of contrasting them was to identify *whether* and *how* the neurophysiological parameters considered changed owing to the presence of stressful events, since the potential contributions of other mental states, such as workload, mutually elide with one other. The four slots were in fact designed to differ mainly by the presence and level of induced stress (Fig. 1). In the second phase of the analysis, the neurophysiological parameters were merged and employed as a features set for the *stepwise linear discriminant analysis* (SWLDA)^100,101^ algorithm with the aim of investigating whether considering all of them at the same time could provide a more accurate and reliable measurement of stress. A summary description of the various analyses is given in Table 1, whilst their systematic and detailed descriptions are set out in the following sections.

**Table 1:** Summary of the analyses.

Aim of the analysis	Actions
Identifying *whether* and *how* the neurophysiological parameters considered changed owing to the presence of stressful events	Wilcoxon signed-rank tests on the averaged values of the PSD, HR, LF/HF, SCL, and SCR by comparing the no-stress (SLOT#1) and high-stress (SLOT#3) ATM phases.
Definition of single-parameter stress indexes	Depending on the previous results, the neurophysiological parameters which indicated significant changes under the high-stress ATM phase were employed individually to define stress indexes, and their capability to assess stress levels over the entire ATM scenario was then estimated by the Friedman analysis.
Definition of the Fusion-based stress index	All the previous neurophysiological parameters were merged and employed as a feature set for the SWLDA to find out if considering all of them at the same time could provide a more accurate and reliable measurement of stress. A Friedman analysis was performed on the Fusion-based stress index over the entire ATM scenario.

### Identification of the most sensitive neurophysiological parameters

Several studies have indicated that mental workload and stress are interconnected and that their measurement could be influenced by one other^102^. More generally speaking, most experimental protocols and laboratory tasks may involve and affect more than one mental state at a time (for example mental workload, stress, attention, vigilance, drowsiness, mental fatigue), as it can be very difficult, sometimes even impossible, to achieve complete and total control of the phenomenon being investigated. One possible solution, especially under realistic settings and while real working activities are being carried, as in our case, could be to emphasise the phenomenon being considered (i.e. stress) as far as possible by defining similar experimental conditions differing mainly by the presence/absence of the investigated cognitive phenomenon and with different intensities (e.g. *no stress*, *medium stress*, *high stress*). In our case, to identify which neurophysiological parameters varied significantly under high stress (step#1), we compared the ATM phase with no stressful events, i.e. SLOT#1, with the ATM phase with the high-stress events and the same sequence of workload levels (easy → medium → hard), i.e. SLOT#3. As stated above (see *the* paragraph headed *Air traffic management simulation*), the contrast between these two phases allowed us to mutually elide, or at least mitigate, the potential contributions of other factors, such as workload, and emphasize the impact of stress. Additionally, another purpose of this analysis was to avoid approaches in which all the available features were used to train the algorithm without considering the physiological meaning of the features themselves (i.e. blind selection)^28,103^. The consequence is in fact likely to be a definition of models and classification accuracy resulting from confounds such as features not strictly linked to the cognitive phenomenon being investigated (for example non-removed artifacts or other mental states) or isolated events (for example specific ATC events within the experimental tasks). The outcomes of this analysis provided the most sensitive brain and autonomic parameters for stress, and they were consequently used to define the *single-parameter* and *Fusion-based* stress indexes.

### Single-parameter stress index definition

The autonomic parameters identified in step#1 were averaged within each ATM slot (SLOT#1-SLOT#4) to define the *ECG-* and *GSR-*based stress index. In particular, for each controller we obtained four values for the GSR and ECG parameters (e.g. SCL, and LF/HR), and then Friedman analyses were performed on those values, separately for the *ECG-* and *GSR-*based stress index, to assess their capability of discriminating the different stress levels over the entire ATM scenario. For the *EEG-*based stress index, the PSD features derived in step#1 were used as a feature set for the SWLDA algorithm^104^, and then cross-validations were performed as described in the paragraph headed *Stress condition discrimination and classification accuracy*. The outputs of the SWLDA, i.e. the linear discriminant function *y(t)*, were finally averaged across the cross-validations to define the EEG-based stress index, and then a Friedman analysis was performed on it as for the other two single-parameter stress indexes.

### Fusion-based stress index definition

The final set of neurophysiological parameters was finally merged to define the *Fusion-based* stress index. In particular, the features were merged every 30 s with a moving window of 0.5 s and for each controller, in accordance with the results of the step#1 analyses, the final features vector consisting of 58 features: 1 LF/HF, 1 SCL, 56 PSDs (14 EEG channels * 4 EEG bands). In total, each controller had about 7,140 labelled feature vectors over the entire ATM scenario (((60 min × 60 s) − 30 s)/0.5 s). Those feature vectors were then employed as a training and testing dataset for the SWLDA as described in the paragraph headed *Stress condition discrimination and classification accuracy*. The linear discriminant functions were averaged across the cross-validations to define the Fusion-based stress index, and finally the Friedman analysis was performed to investigate whether considering all the neurophysiological features at the same time could provide a more accurate and reliable stress assessment than taking them individually.

### Data fusion

Data fusion can take place at four different levels: *signal-level fusion* (direct or raw data fusion), *pixel-level fusion* (for image data), *feature-level fusion*, and *symbol-level fusion*^105^. Signal-level fusion can be applied to combine directly commensurate data. For data which is non-commensurate, fusion takes place at feature level. In particular, features are usually extracted from the sensors and used to form a *feature vector* which, after fusion, will result in a higher level representation of the data^106,107^. Since the neurophysiological signals considered (i.e. EEG, ECG, and GSR) were non-commensurate, the *feature fusion* criterion was adopted in this work. Once the neurophysiological features (for example PSD, SCL, SCR, HR, and LF/HR) had been estimated, two tasks were carried out before merging the features: *data normalisation* and *data resampling*^108^. These operation are generally necessary because the range of values of the raw data might vary widely, the different feature datasets could be unbalanced owing to the rejected epochs, and the machine-learning algorithm considered might not work properly under such conditions. In this work, the method used for normalisation was z-score transformation^109^. For the resampling task, the *synthetic minority oversampling technique* (SMOTE) was applied. As described in detail by Chawla^76^, the SMOTE is an oversampling approach in which the minority class is oversampled by creating “synthetic” examples rather than by oversampling with replacement. Depending on the amount of oversampling required, neighbours from the *k*-nearest neighbours are randomly chosen. In our work, we set *k* = 5.

### Stress condition discrimination and classification accuracy

In order to avoid bias in the stress assessment owing to different workload demands, we performed three cross-validations by dividing the entire ATM scenario into four 15-minute slots (SLOT#1-SLOTS#4), each of them with a different stress level (*no stress*, *medium stress*, *high stress*). Within each slot, three 5-minute workload conditions were identified, each of them corresponding to three levels (LOW, MEDIUM, HIGH) for a total of twelve 5-minute phases designated LWL1, MWL1, HWL1, LWL2, MWL2, HWL2, LWL3, MWL3, HWL3, LWL4, MWL4 and HWL4 (Fig. 1). As stated above, there were no stressful events in SLOT#1 or SLOT#4, and they were therefore designated *low-stress* phases. In SLOT#2 (*medium stress*), there were three 20-second medium-stressful events (SM1, SM2, and SM3 – orange circles in Fig. 1), while in SLOT#3 (*high stress* – red circles in Fig. 1), there were three 20-second high-stress events (SH1, SH2, and SH3). The three cross-validations consisted in training the SWLDA algorithm with the corresponding 5-minute LOW and HIGH workload conditions of SLOT#1 and SLOT#3, and then testing it on the remaining ones. In other words, we trained the SWLDA with phases LWL1-LWL3 (i.e. low workload with low-stress, and low workload with high-stress), and tested it on phases MWL1, HWL1, LWL2, MWL2, HWL2, MWL3, HWL3, LWL4, MWL4 and HWL4. Similarly, we used phases MWL1-MWL3 and HWL1-HWL3 to train the SWLDA, and then the remaining ones to test the corresponding models. For each cross-validation and stress index, the *area under curve* (AUC) and *classification accuracy* (ACC)^110^ were estimated and finally averaged to obtain overall values corresponding to the *no stress* vs *medium stress*, *no stress* vs *high stress* and *medium stress* vs *high stress* comparisons. The rationale was to define a model able to identify the most significant features “purely” linked to stress. Finally, for each stress comparison pair, the AUC and ACC distributions obtained from the experimental data (*Measured*) were compared with random distributions (*Random*) estimated by shuffling the stress condition labels and then averaging the resulting AUC and ACC values for each possible comparison. The aim was to determine whether the stress classification was due to chance^28^, i.e. to assess the reliability of the stress index considered. In conclusion, as suggested by Lobo *et al*.^111^, we reported both the AUC and ACC measurements so that the relative importance of errors of commission and omission could be considered in order to assess the performance of the proposed method. Logistic regression in fact returns positive/negative values depending on whether the logistic function is greater/smaller than a threshold, usually 0.5 by default. When we choose a threshold (i.e. a cut-point), we have a classifier. For a given choice of threshold, we can compute the ACC, which is the proportion of true positives (TP) and negatives (TN) in the whole dataset. The AUC on the other hand measures how the true positive rate (recall) and the false positive rate trade off, and thus the AUC is not a function of the threshold, since it is an evaluation of the classifier, as the threshold varies over all possible values. In addition, the AUC has a different interpretation. It is also the probability that a randomly chosen positive example is ranked above a randomly chosen negative example, in accordance with the classifier’s internal value for the examples. In other words, the ACC is based on a specific threshold, whereas the AUC tries all the thresholds.

### Statistical analysis

Since all the data distributions were not normally distributed (i.e. they were Gaussian), we could not employ parametric statistical tests, but ad to apply non-parametric ones instead. In particular:

#### Subjective data

The Friedman analysis (α = 0.05) was performed on the stress perception and efficiency ratings (*within* factor: ATM Phases; 4 levels: SLOT#1-SLOT#4) to assess how the ATCOs and SMEs perceived the impact of the stressful events throughout the ATM scenario. In addition, repeated correlation analysis was performed between the ATCO and SME stress scores to determine possible differences in terms of stress perception.

#### Neurophysiological data

Wilcoxon signed-rank tests (α = 0.05) were performed between the SLOT#3 (high-stress condition) and SLOT#1 (no-stress condition) phases on each of the neurophysiological parameters in order to identify the most sensitive features for the definition of the mathematical stress model.

#### Stress indexes

Friedman analyses (α = 0.05) were performed on the different stress indexes (*within* factor: ATM Phases; 4 levels: SLOT#1-SLOT#4) to evaluate their capabilities in assessing the different stress levels throughout the ATM scenario. In addition, repeated correlation analysis was performed between the stress indexes and ATCO efficiency scores to determine any correlation between the neurophysiological and subjective measurements.

#### Classifier data

Friedman analyses (α = 0.05) were performed on the averaged AUC and ACC values (*within* factor: cross-validations; three levels: n*o stress* vs *medium stress*, *no stress* vs *high stress* and *medium stress* vs *high stress* comparisons) to evaluate the capability of the stress index in terms of discrimination and classification. Finally, the *Measured AUC (ACC)* and *Random AUC (ACC)* distributions were compared using Friedman analyses (α = 0.05) to assess the reliability of the stress index considered.

## Results

### Subjective stress perception

**Figure 3:**
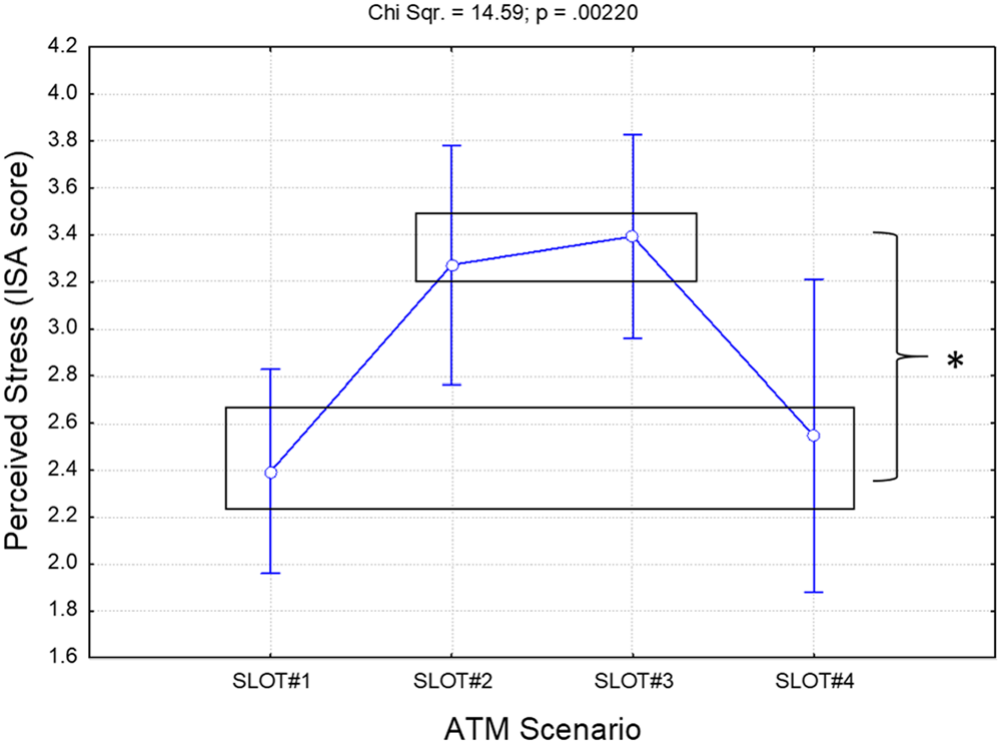
Results of the Friedman analysis of the self-reported stress perception provided by the ATCOs while dealing with the ATM scenario. The black boxes group the conditions exhibiting the same statistical differences with respect to the others. In particular, both SLOT#2 and SLOT#3 proved the most stressful, while SLOT#1 and SLOT#4 were the least stressful. The asterisk means that the differences were statistically significant (p < 0.05).

The Friedman analysis of the self-reported stress perception provided by the ATCOs every five minutes while dealing with the ATM scenario showed a significant effect (Chi Sqr. = 14.59; p = 0.0022) throughout the ATM scenario (SLOT#1-SLOT#4). In particular, the Bonferroni post-hoc tests showed how both SLOT#2 and SLOT#3 produced the most stressful phases, while SLOT#1 and SLOT#4 produced the least stressful ones (Fig. 3).

**Figure 4:**
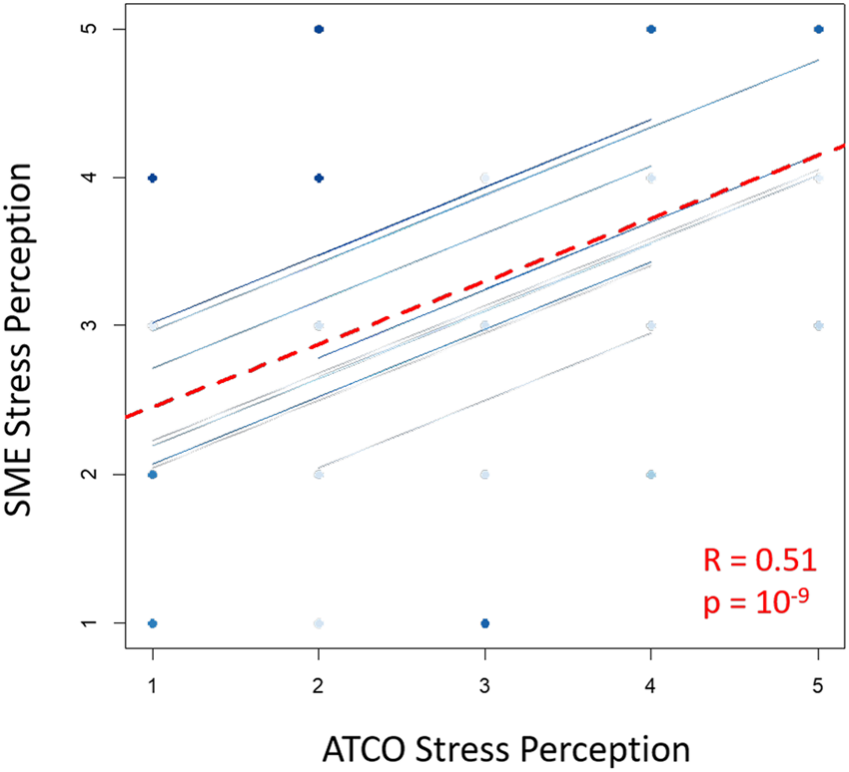
Scatterplot of the repeated measure correlation analysis between the ratings provided by the ATCOs (x axis) and those of the SMEs (y axis) as a subjective measure of the stress demand throughout the execution of the ATM scenario. The high (R = 0.51) and significant (p < 0.001) correlation demonstrated how both ATCOs and SMEs had the same perception of stress demand. The dashed red line represents the averaged correlation, while the solid blue lines indicate the correlations for each controller and the corresponding SME who sat behind him.

Repeated measures correlation analysis was then performed between the ISA scores provided by the SMEs and ATCOs at the same time instants throughout the execution of the ATM scenario. The results highlighted a high (R = 0.51) and significant (p < 0.001) correlation between the two subjective measurements (Fig. 4), demonstrating how both the controllers (ATCOs) and the external supervisors (SMEs) exhibited the same stress perception.

### ATCO efficiency assessment

**Figure 5:**
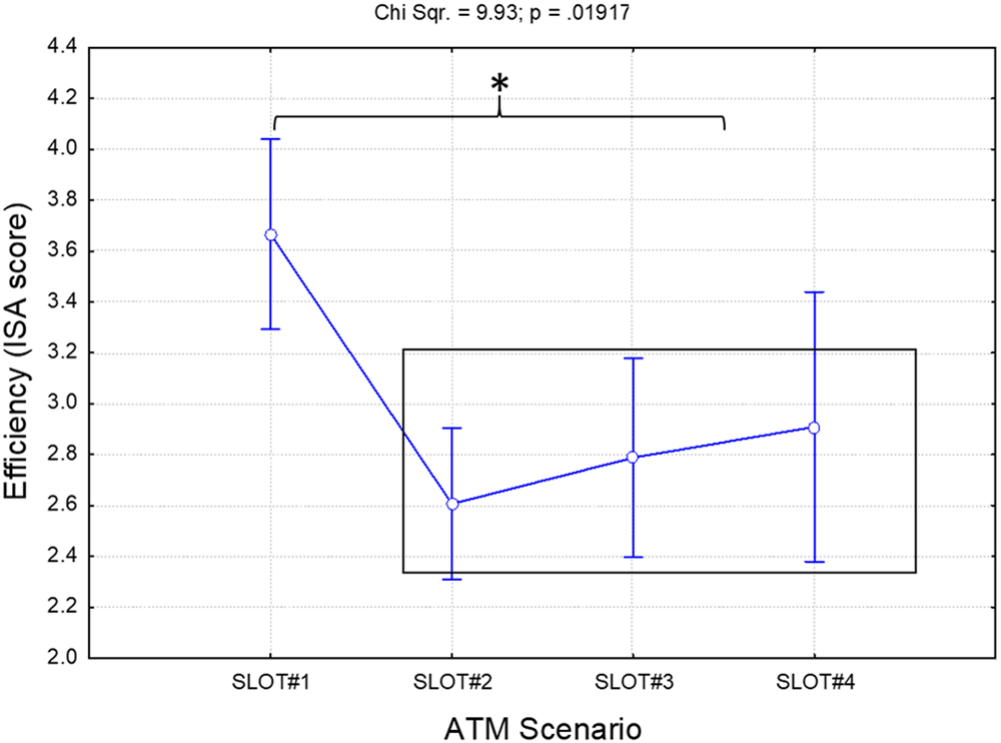
Results of the Friedman analysis of the ATCO’s efficiency score provided by the SMEs throughout the ATM scenario (SLOT#1-SLOT#4). The black box groups the conditions exhibiting the same statistical differences with respect to the other. In particular, Efficiency was high in SLOT#1 but then dropped off significantly (all p < 0.004) from the beginning of the stressful events (SLOT#2) to the end of the simulation (SLOT#4). The asterisk means that the differences were statistically significant (p < 0.05).

The Friedman analysis on the ATCO efficiency scores provided by SMEs every five minutes showed a significant effect (Chi Sqr. = 9.93; p = 0.019) throughout the execution of the ATM scenario. In particular, the efficiency was high in SLOT#1 (Fig. 5) but then dropped off significantly (all p < 0.004) from the beginning of the stressful events (SLOT#2) to the end of the ATM simulation (SLOT#4).

### Impact of stress on brain activity

**Figure 6:**
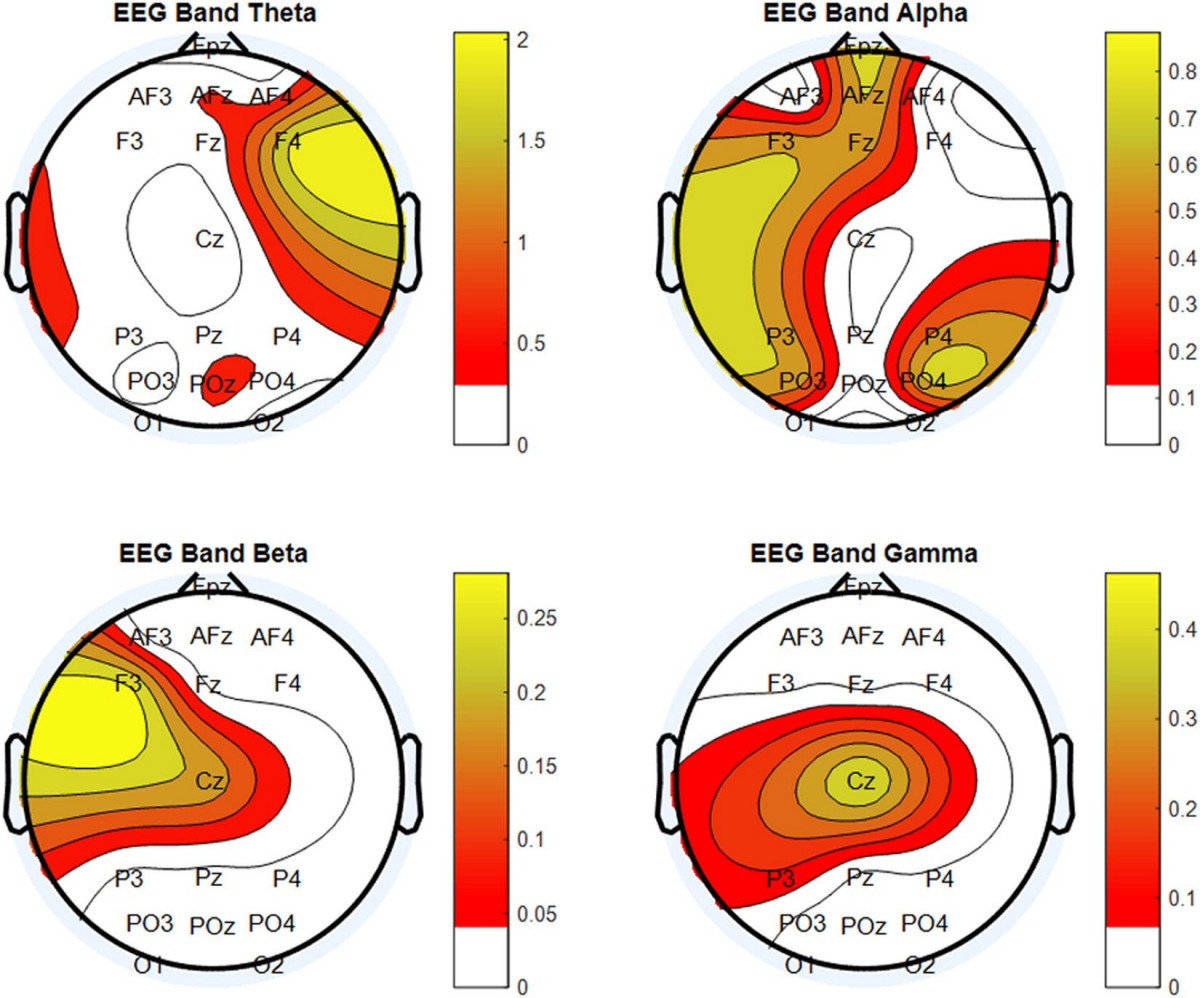
Results of the Wilcoxon signed-rank test on the averaged PSDs in the considered EEG bands (theta, alpha, beta, and gamma). The test indicated a significant PSD increment (all p < 0.05) over the frontal, and parieto-occipital brain areas in all the frequency bands (yellow and red colours) when stress was high (SLOT#3). The EEG channels were left blank when no statistical difference was found between the low (SLOT#1) and high stress (SLOT#3) condition.

The Wilcoxon signed-rank tests between SLOT#3 and SLOT#1 showed how the stressful events caused significant EEG PSD increments (Fig. 6). In particular, significant PSD changes (all p < 0.05) were found over the frontal, and parieto-occipital brain areas (yellow and red coloured areas) in all the EEG frequency bands considered, i.e. theta, alpha, beta and gamma. These brain areas were then used as a feature domain for the definition of the stress model by the SWLDA.

### Impact of stress on the autonomic signals

**Figure 7:**
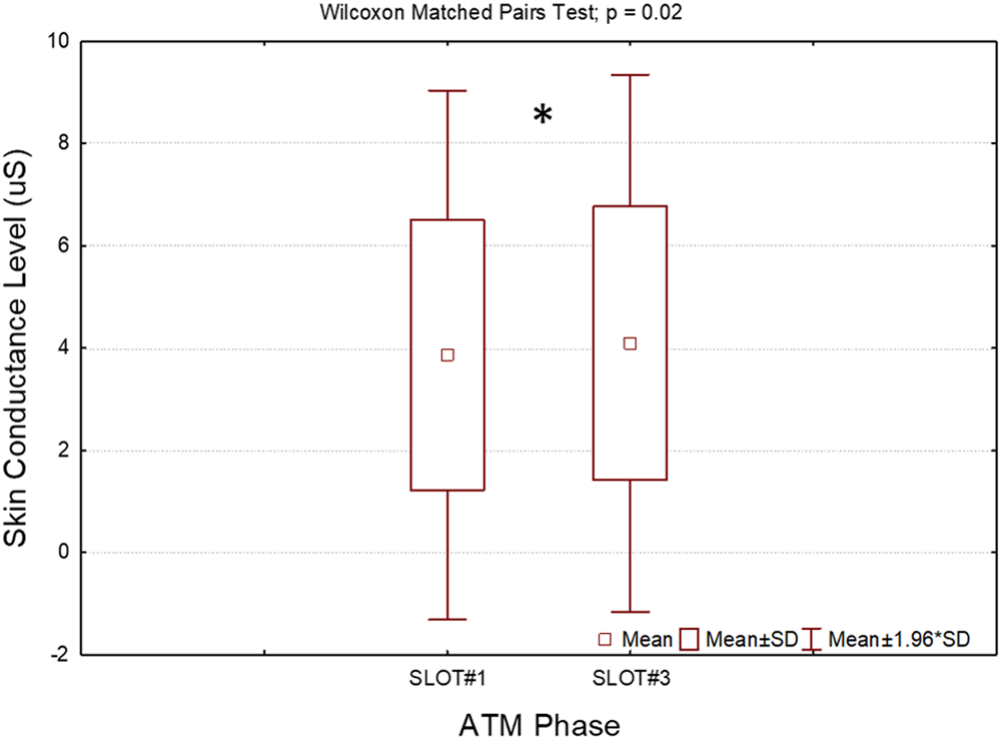
Results of the Wilcoxon signed-rank test on the averaged SCL component derived from the GSR analysis. The SCL indicated a significant increment (p = 0.02) during the high-stress phase (SLOT#3) with respect to the ATM phase with no stressful events (SLOT#1). The asterisk means that the differences were statistically significant (p < 0.05).

The Wilcoxon signed-rank tests on the GSR components (i.e. SCL and SCR) between SLOT#1 and SLOT#3 indicated significant differences (p = 0.02) only for the SCL component. In particular, the SCL assumed higher values during the high-stress phase (Fig. 7) than during the ATM phase with no stressful events (SLOT#1), and it was therefore included in the feature set for the definition of the stress model. The SCR on the other hand did not indicate any significant changes between the two conditions (p = 0.12).

**Figure 8:**
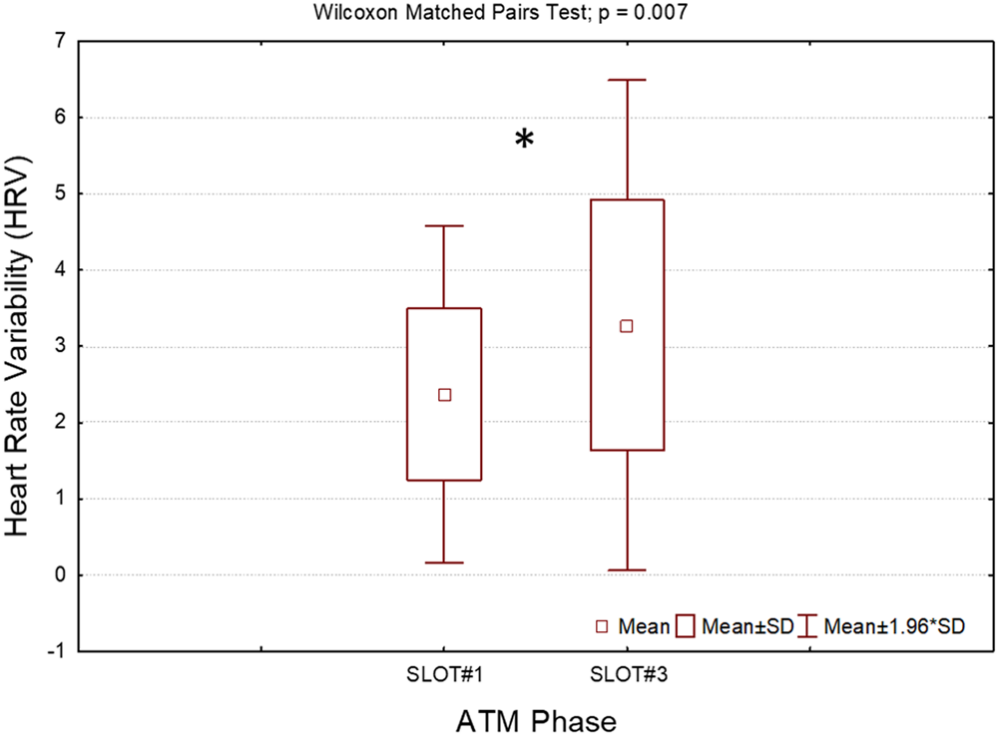
Results of the Wilcoxon signed-rank test on the averaged LF/HF ratio derived from the HR spectral analysis. The LF/HF indicated a significant increase (p = 0.007) during the high-stress phase (SLOT#3) with respect to the ATM phase with no stressful events (SLOT#1). The asterisk means that the differences were statistically significant (p < 0.05).

Similarly, the Wilcoxon signed-rank tests on the ECG parameters (i.e. HR and LF/HF) between SLOT#1 and SLOT#3 indicated a significant increment (p = 0.007) in the LF/HF during the high-stress phase (Fig. 8), whilst the HR did not indicate any statistical changes between the two ATM phases considered (p = 0.22). The LF/HF was thus added to the feature set for the definition of the stress model together with the PSD and SCL parameters.

### EEG-based stress index

**Figure 9:**
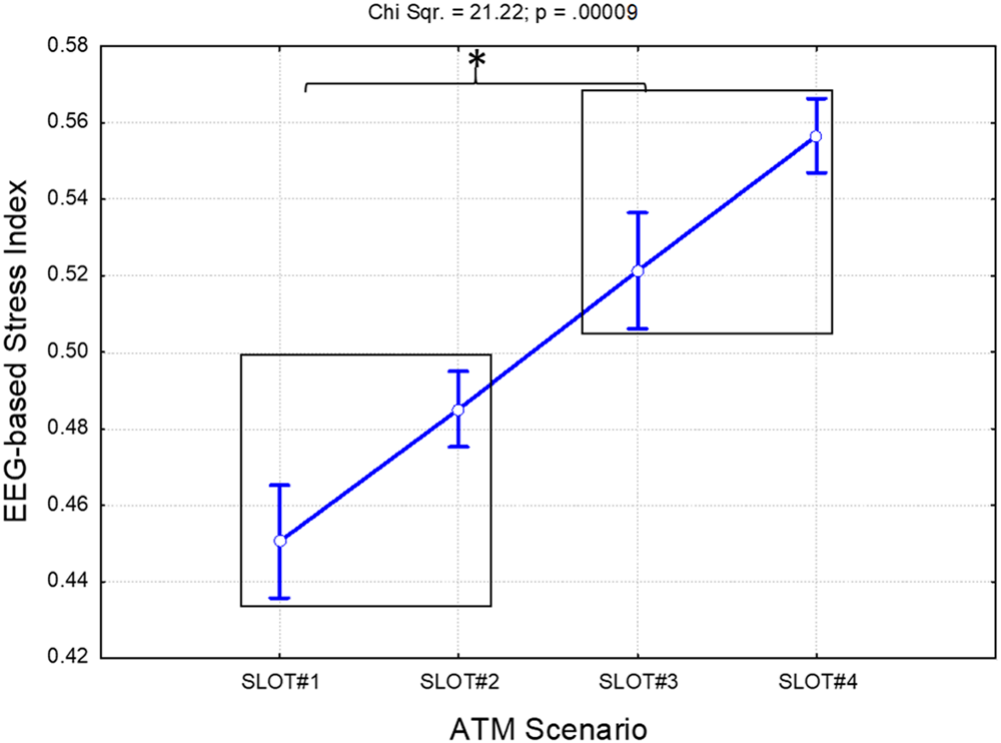
Results of the Friedman analysis of the EEG-based stress index throughout the ATM scenario. The black boxes group the conditions exhibiting the same statistical differences with respect to the others. In particular, the EEG-based stress index indicated a significant increment (all p < 0.0009) within the SLOT#3-SLOT#4 phases with respect to the SLOT#1-SLOT#2 ones. The asterisk means that the differences were statistically significant (p < 0.05).

The Friedman analysis of the stress index based only on the EEG features (the *EEG-based stress index*) indicated a significant effect (Chi Sqr. = 21.22; p < 0.001) throughout the ATM scenario. In particular, the Bonferroni post-hoc tests highlighted significant increments (all p < 0.001) in the EEG-based stress index in both the SLOT#3 and SLOT#4 phases with respect to the SLOT#1-SLOT#2 phases (Fig. 9).

### ECG-based stress index

The Friedman analysis of the stress index based only on the ECG features (i.e. average LF/HF values) did not indicate any significant changes (p = 0.25) throughout the ATM scenario.

### GSR-based stress index

The Friedman analysis of the stress index based only on the GSR features (i.e. average SCL values) did not indicate any significant changes (p = 0.08) throughout the ATM scenario.

### Fusion-based stress index

**Figure 10:**
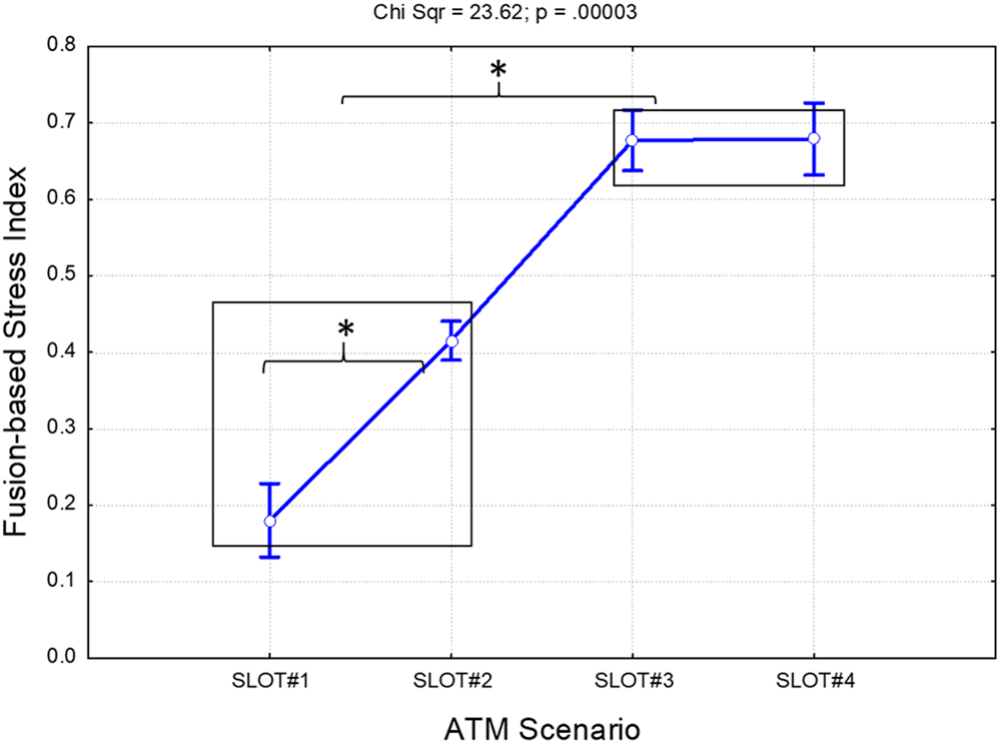
Results of the Friedman analysis of the Fusion-based stress index. A significant effect (p < 0.001) was found throughout the ATM scenario. The black boxes group the conditions exhibiting the same statistical differences with respect to the others. In particular, the Fusion-based stress index kept increasing, indicating significant (all p < 0.02) variations from SLOT#1 to SLOT#3, and finally reaching a plateau exhibiting no difference (p = 1) between SLOT#3 and SLOT#4. The asterisk means that the differences were statistically significant (p < 0.05).

In order to find out whether considering both the cognitive and hormonal aspects of the stress responses simultaneously could improve the measurement of stress itself, all the previous features (i.e. PSD, LF/HF and SCL) were merged, as described above, to define the *Fusion-based stress index*. Figure 10 shows the results of the Friedman analysis of the Fusion-based stress index throughout the ATM scenario. The statistical analysis reported a significant effect (Chi Sqr. = 23.62; p < 0.001) across the slots. In particular, the Bonferroni post-hoc tests revealed significant differences (all p < 0.02) among all the ATM phases considered (SLOT#1-SLOT#4) except for SLOT#3 and SLOT#4, i.e. during the last 30 minutes. In other words, the level of stress kept increasing from SLOT#1 to SLOT#3, and finally did not change (p = 1) between SLOT#3 and SLOT#4, revealing how the effect of the stressful events probably lasted even after the events themselves were over.

**Figure 11:**
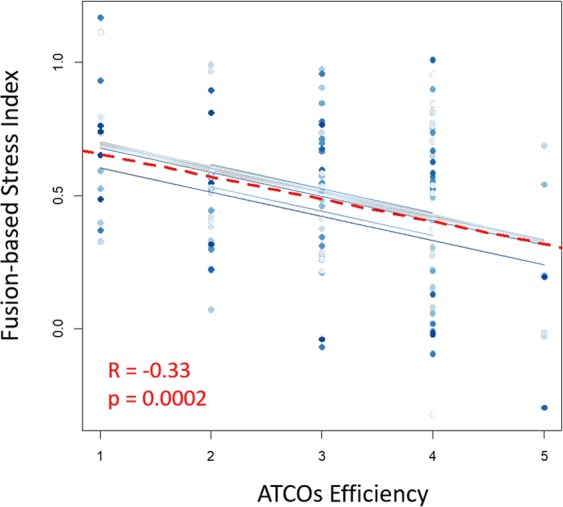
Scatterplot of the repeated measure correlation analysis between the ATCOs’ efficiency (x axis) ratings provided by the SMEs during the execution of the ATM scenario and the Fusion-based stress index (y axis). The results reported an inverse (R = −0.33) and significant (p < 0.001) correlation between the two measures. The dashed red line represents the averaged correlation, while the solid blue lines indicate the correlations for each controller’s efficiency and the corresponding Fusion-based stress index.

The repeated measure correlation analysis between the Fusion-based stress index and the efficiency scores exhibited inverse (R = −0.33) and significant (p < 0.001) correlation between the two measurements, demonstrating the negative impact of stressful events on user performance (Fig. 11).

The same correlation analysis was performed between the EEG-based stress index and the efficiency scores, but no correlation (R = 0.05; p = 0.53) was found. For this reason, the AUCs and ACCs were calculated only for the Fusion-based stress index and they are reported in the following paragraph.

### Stress condition discrimination and classification

**Figure 12:**
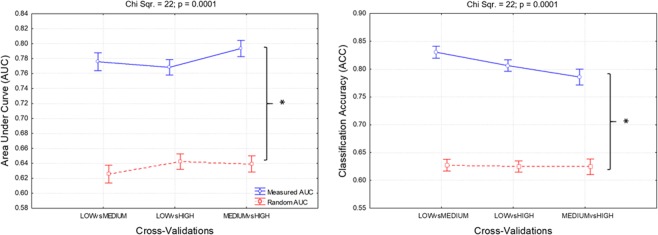
Results of the Friedman analysis of the averaged AUCs and ACCs. In particular, the measured AUC and ACC (blue lines) were significantly higher (p < 0.001) than the random ones (random AUC and ACC, red lines), demonstrating the reliability of the Fusion-based stress index in terms of stress discrimination and classification. The asterisks mean that the differences were statistically significant (p < 0.05).

The Fusion-based stress index reported both a consistent trend with the scientific literature (i.e. the effect of stressful events lasted over time), and a strong capability to assess the impact of stress on ATCO efficiency throughout the ATM scenario (i.e. stress increments with the occurrence of stress events and performance degradation). In order to quantify this capability and reliability, the discrimination (AUC) and classification (ACC) accuracies of the three stress levels (*low*, *medium*, *and high*) were calculated. Figure 12 shows the *Measured AUCs* (blue line in the left panel) and *ACCs* (blue line in the right panel) with respect to the *Random AUCs* (red line in the left panel) and ACCs (red line in the right panel). The statistical analysis indicated significant (all p < 0.001) differences between them, and good reliability in terms of stress level discrimination (AUC) and classification (ACC) as all the averaged *Measured AUCs* were greater than 0.7 (left panel), and the *Measured ACCs* were all somewhat greater than 0.8 (right panel).

## Discussion

Generally, when users are under prolonged stressful conditions, or after high-stress events, the capability to properly evaluate a situation is likely to be affected by the stress, and they may be prone to *over-* or *under-*estimate the stress level experienced. By employing neurophysiological measurements, which do not depend on the user’s feeling, perception, or past experiences, we can overcome such limitations and better characterise and assess stress changes while users dealing with tasks. In addition, since hormonal processes are likely to take longer to return to normal activations than cognitive processes, the effects of stressful events will result in a transient effect over time even once the stressful events are over.

The results of our work have highlighted three important key points for stress evaluation in ATM contexts. The first key aspect is that the designed stressful events significantly affected both the efficiency and the psychophysiological status of the controllers. The ATCOs’ efficiency in fact decreased significantly (p = 0.019) from the beginning of the stressful events (SLOT#2) as reported in Fig. 5. In addition, inverse (R = −0.33) and significant (p < 0.001) correlation was found between the Fusion-based stress index and the ATCOs’ efficiency as reported by the SMEs (Fig. 11). In other words, these results showed how high-stress events kept the controllers reacting in terms of both their cognitive and their hormonal response, and at the same time significantly affected performance, and hence overall safety.

The second key aspect is related to the importance and advantages of employing neurophysiological measurements instead of subjective ones. In this regard, the results derived from the stress perception ratings demonstrated how both the controllers and SMEs were not able to recognise, and consequently evaluate, the impact of stress once the stressful events were over (SLOT#4). Both groups in fact rated the stress experienced as “low” (Figs. 3 and 4) during the last phase (SLOT#4), while both the *EEG-* and *Fusion-based* stress indexes showed how the stress kept increasing significantly (Figs. 9 and 10) from the beginning of the stressful events (SLOT#2) to the end of the ATM scenario (SLOT#4).

Although the controllers were convinced that they were experiencing low-stress conditions and did not realise the significant drop in their efficiency, the transient effect exhibited by the Fusion-based index between SLOT#3 and SLOT#4 is well supported by and consistent with previous stress-related studies^4,9,19^.

The third key aspect concerns the choice of measurements to properly assess the *stress* experienced by the controllers. In this regard, we considered the EEG signal as an indicator of the cognitive processes^35,36^, and the ECG (i.e. LF/HF parameter) and GSR (i.e. SCL component) as indicators of the hormonal reactions, i.e. glucocorticoid and catecholamine release^38–40,112^. The *Fusion-based stress index* revealed higher capability in discriminating and classifying the different stress levels (Fig. 10) than the other indexes (Fig. 9) throughout the ATM scenario considered. Both the Measured AUCs and ACCs (Fig. 12) assumed higher values than the Random ones (all p < 0.001), demonstrating the advantages of considering both the cognitive and hormonal processes underlying user behaviour stress measurement.

Since stress-related scientific literature and physiology have widely demonstrated how the effects of stressful events can last over time, it is not possible to predict or keep under control the stress-related transient durations of different users^113^. As a consequence, it was not possible to randomise the stressful events and phases among the controllers. It is therefore important to emphasise that our conclusions are mainly based on the correlation between the Fusion-based stress index and the Efficiency measurement, and that there was no correlation between the subjective and efficiency measurements, i.e. the controllers were convinced that they were not stressed at the end (SLOT#4), although their performance did not recover the value exhibited in the previous no-stress phase (SLOT#1). However, owing to the trend in the proposed stress measurement, we deduced a certain relationship between the Fusion-based stress index and the stress experienced by the controllers, thus suggesting that it can be used in larger and differently designed experiments. Despite the promising and innovative results, the small experimental group of sixteen controllers and the execution of only one ATM scenario will in fact prompt us to increase the number of participants and design more ATM scenarios to further validate and assert the evidence presented. In addition, we will further investigate the difference between the user’s stress perception and the neurophysiological measurements by evaluating other aspects such as *arousal*, *frustration*, and *motivation* in order to determine whether and how they contribute to the definition of the stress index.

It should, however, be noted that our work highlights the usefulness and importance of employing neurophysiological measurements and a multimodal approach for the purposes of accurate stress assessment, especially while dealing with high-risk tasks^32,114^.

## Conclusions

Studies related to stress assessment usually aim only to differentiate stress levels in the experimental conditions considered without considering the potential impact and effect of acute stressful events on the cognitive and hormonal aspects over time. The scientific literature provides many studies and evidence of transient effects due to high-stress events, and if this aspect is not taken into account, the results of a study might be misinterpreted or underestimated. The evidence identified in our work suggest that combination of the EEG, ECG and GSR make it possible to define a stress index capable of characterising variations in stress experienced while dealing with realistic ATM activities, and provide a reliable measurement of stress, thereby overcoming many limitations of conventional measurements such as self-reporting. Perceived stress was in fact different from that revealed by the neurophysiological measurements. Furthermore, for the purposes of the definition of the mathematical stress model, we did not apply a straight blind selection procedure. We firstly identified which neurophysiological features changed significantly from a *no-stress* to a *high-stress* condition, and then we employed that feature set for the definition of the stress model by the SWLDA.

The purpose of our approach was both to provide a set of features mainly linked to stress, therefore taking into account their neurophysiological meaning, and then to allow the machine-learning algorithm to select the most significant features, rather than choosing among all the available data with the risk of confounds due to features not strictly linked to the phenomena being investigated, or to specific events within the experimental tasks. In conclusion, we have demonstrated the importance of the *points in time* at which stress is measured (i.e. the impact of stressful events will probably last even once the events themselves are over), and of the *choice of metrics* used to accurately and reliably assess the stress experienced by users (i.e. the advantages of the neurophysiological signal and multimodal approach).
